# Direct cellular reprogramming enables development of viral T antigen–driven Merkel cell carcinoma in mice

**DOI:** 10.1172/JCI152069

**Published:** 2022-04-01

**Authors:** Monique E. Verhaegen, Paul W. Harms, Julia J. Van Goor, Jacob Arche, Matthew T. Patrick, Dawn Wilbert, Haley Zabawa, Marina Grachtchouk, Chia-Jen Liu, Kevin Hu, Michael C. Kelly, Ping Chen, Thomas L. Saunders, Stephan Weidinger, Li-Jyun Syu, John S. Runge, Johann E. Gudjonsson, Sunny Y. Wong, Isaac Brownell, Marcin Cieslik, Aaron M. Udager, Arul M. Chinnaiyan, Lam C. Tsoi, Andrzej A. Dlugosz

**Affiliations:** 1 Department of Dermatology,; 2 Department of Pathology,; 3 Michigan Center for Translational Pathology,; 4 Rogel Cancer Center, and; 5 Department of Computational Medicine and Bioinformatics, University of Michigan, Ann Arbor, Michigan, USA.; 6 Department of Cell Biology, Emory University School of Medicine, Atlanta, Georgia, USA.; 7 Department of Internal Medicine, University of Michigan, Ann Arbor, Michigan, USA.; 8 Department of Dermatology and Allergy, University Medical Center Schleswig-Holstein, Kiel, Germany.; 9 A. Alfred Taubman Medical Research Institute and; 10 Department of Cell & Developmental Biology, University of Michigan, Ann Arbor, Michigan, USA.; 11 Dermatology Branch, National Cancer Institute, Bethesda, Maryland, USA.; 12 Howard Hughes Medical Institute,; 13 Department of Urology, and; 14 Department of Biostatistics, Center for Statistical Genetics, University of Michigan, Ann Arbor, Michigan, USA.

**Keywords:** Dermatology, Oncology, Mouse models, Oncogenes, Skin cancer

## Abstract

Merkel cell carcinoma (MCC) is an aggressive neuroendocrine skin cancer that frequently carries an integrated Merkel cell polyomavirus (MCPyV) genome and expresses viral transforming antigens (TAgs). MCC tumor cells also express signature genes detected in skin-resident, postmitotic Merkel cells, including atonal bHLH transcription factor 1 (*ATOH1*), which is required for Merkel cell development from epidermal progenitors. We now report the use of in vivo cellular reprogramming, using ATOH1, to drive MCC development from murine epidermis. We generated mice that conditionally expressed MCPyV TAgs and ATOH1 in epidermal cells, yielding microscopic collections of proliferating MCC-like cells arising from hair follicles. Immunostaining of these nascent tumors revealed p53 accumulation and apoptosis, and targeted deletion of transformation related protein 53 (*Trp53*) led to development of gross skin tumors with classic MCC histology and marker expression. Global transcriptome analysis confirmed the close similarity of mouse and human MCCs, and hierarchical clustering showed conserved upregulation of signature genes. Our data establish that expression of MCPyV TAgs in ATOH1-reprogrammed epidermal cells and their neuroendocrine progeny initiates hair follicle–derived MCC tumorigenesis in adult mice. Moreover, progression to full-blown MCC in this model requires loss of p53, mimicking the functional inhibition of p53 reported in human MCPyV-positive MCCs.

## Graphical abstract

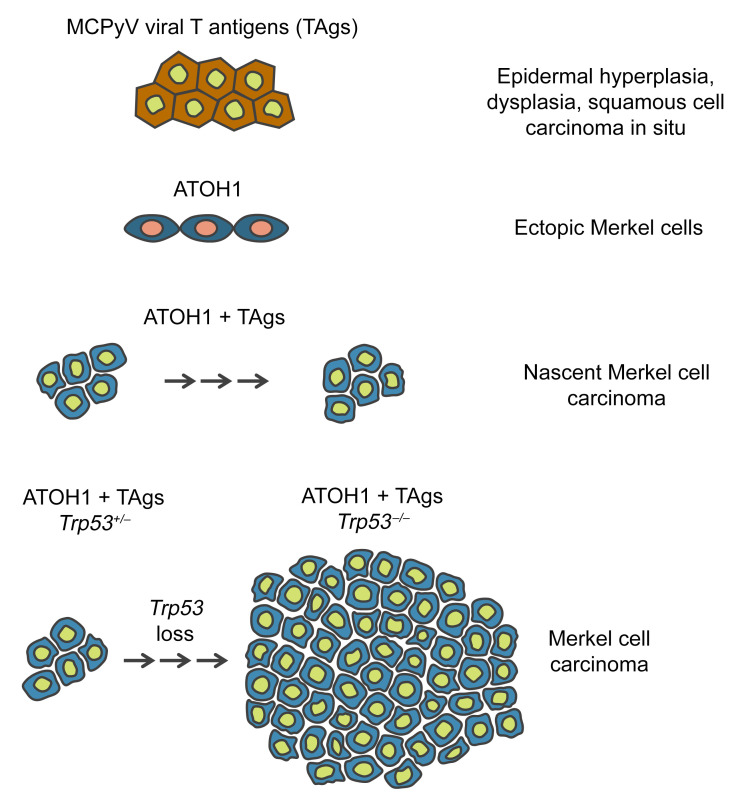

## Introduction

Merkel cell carcinoma (MCC) is an aggressive neuroendocrine skin tumor with a poor prognosis (1), although up to 50% of patients with advanced disease respond to immunotherapy (2). In 2008, a clonally integrated polyomavirus was discovered in 8 of 10 MCC tumors (3). Subsequent studies confirmed that most MCC tumors contain the integrated Merkel cell polyomavirus (MCPyV) genome and express 2 viral transforming antigens (TAgs), small T (sTAg) and truncated large T (tLTAg) (4, 5). MCPyV-negative MCCs have a high mutation burden with a predominance of UV signature mutations, whereas relatively few mutations are detected in MCPyV-positive MCCs (6–9), arguing that viral TAgs play a central role in virus-positive MCC tumorigenesis. In keeping with this notion, MCPyV TAgs transform cultured cells (10, 11) and are tumorigenic when expressed in vivo (12–16), but a bona fide mouse model of MCC has not been reported despite over a decade of effort by several laboratories.

MCC tumor cells express multiple transcription factors and lineage markers in common with Merkel cells, which are rare, nonproliferative neuroendocrine cells that reside beneath specialized compartments of epidermal cells and transduce light touch and itch sensation to adjacent sensory nerves (17, 18). The cell of origin of MCC is not known (11, 16), hindering efforts to develop a viable mouse model testing the role of MCPyV TAgs in MCC development. However, normal Merkel cells arise from KRT5^+^ epidermal progenitors through the action of atonal bHLH transcription factor 1 (*ATOH1*) (19, 20), and some human MCCs are closely associated with epidermal tumors (21–24), raising the possibility of a common cellular origin. Moreover, ectopic ATOH1 expression can reprogram epidermal cells to form postmitotic Merkel cells in adult mice (25), and we have previously shown that expression of ATOH1 together with MCPyV sTAg yields MCC-like cells in mouse embryos (15). Given the failure of conventional approaches to generate a mouse model of MCC, we set out to ascertain whether ATOH1 could be utilized as a tool to reprogram TAg-expressing epidermal cells into the Merkel cell lineage in adult mice, enabling the development of murine tumors resembling human MCC.

## Results and Discussion

**Figure 1:**
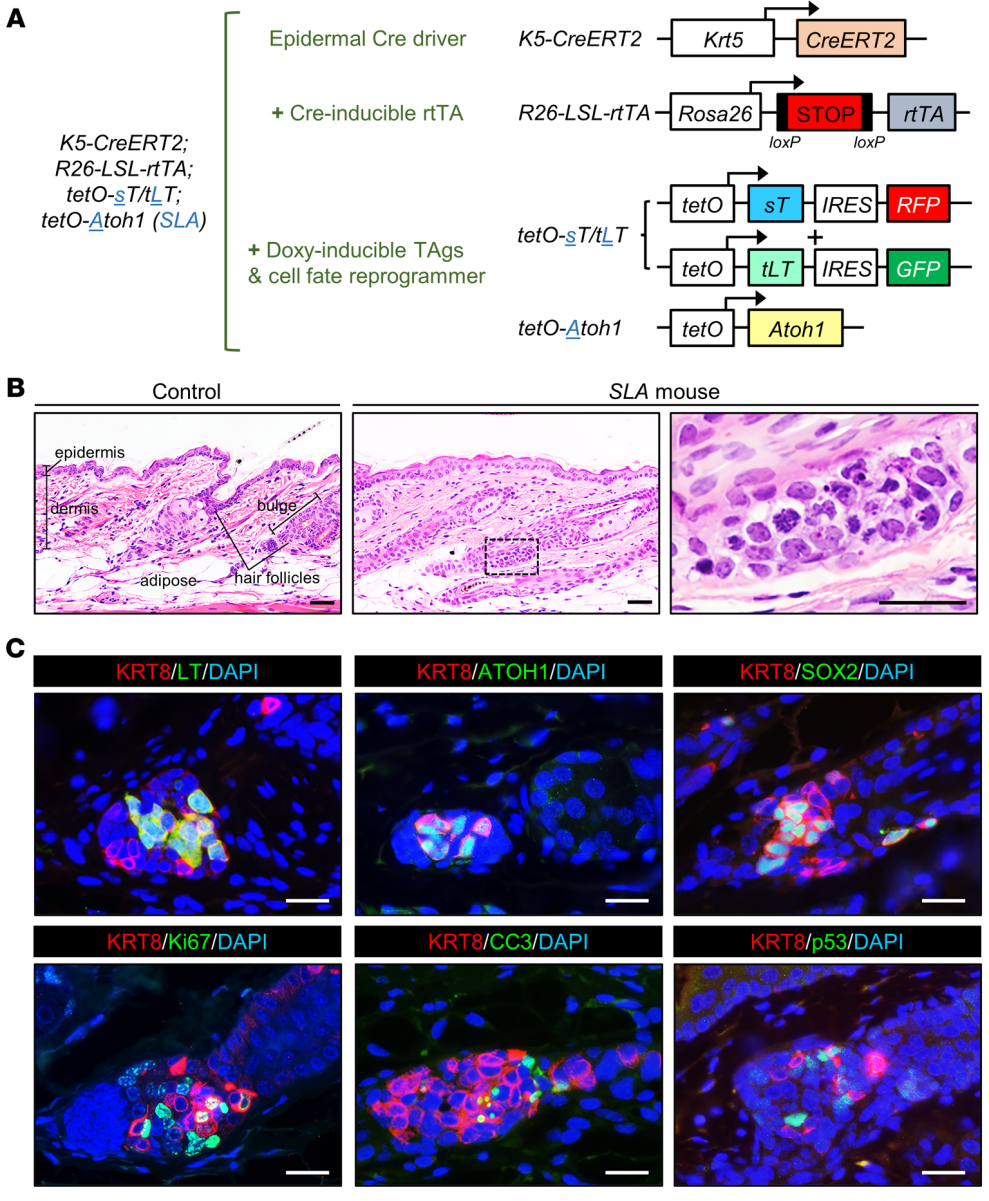
In vivo reprogramming using ATOH1 enables initiation of murine MCC development in mice. (**A**) Combination of mouse strains used to generate *SLA* mice, expressing MCPyV sTAg, tLTAg, and ATOH1, in *Krt5*-expressing cells and their progeny. (**B**) Nascent tumors arising from hair follicle epithelium in *SLA* mice. Scale bars: 50 μm. (**C**) Immunostaining for the indicated markers. Scale bars: 25 μm.

We generated and validated transgenic mouse strains with doxycycline-inducible coexpression of MCPyV sTAg and tLTAg carrying internal ribosome entry site–driven (IRES-driven) red fluorescent protein (RFP) and GFP reporters, respectively (Figure 1A and Supplemental Figure 1; supplemental material available online with this article https://doi.org/10.1172/JCI152069DS1). We next performed crosses with *K5-CreERT2* (26), *R26-LSL-rtTA* (27), and *tetO-Atoh1* mice (28) to generate *K5-CreERT2;R26-LSL-rtTA;tetO-sT/tLT;tetO-Atoh1* mice, which we designated *SLA* (Figure 1A), as well as *SL* mice, which were missing the *tetO-Atoh1* allele (see Methods). Mice were treated with tamoxifen to activate Cre function and rtTA expression and with doxycycline to induce expression of sTAg and tLTAg, with or without ATOH1, in *Krt5*-expressing epidermal cells and their progeny.

Although the *K5-CreERT2* strain drives recombination broadly in the basal layer of hair follicles as well as the interfollicular epidermis (26), examination of tissue sections from TAg-expressing *SL* mice 2.5 weeks or more after transgene induction revealed that LTAg expression became largely restricted to hair follicle epithelium (Supplemental Figure 2). Moreover, histological analysis of sections from *SLA* mice collected 2 weeks after transgene induction revealed spatially restricted, atypical-appearing cellular aggregates near the normally quiescent hair follicle stem cell compartment called the bulge (refs. 29, 30, and Figure 1). The cells in these aggregates contained scant cytoplasm, condensed chromosomes, and pyknotic nuclei (Figure 1B), and they expressed ATOH1, the Merkel cell/MCC markers keratin 8 (KRT8) and SOX2, proliferation marker Ki67, apoptosis marker cleaved caspase-3 (CC3), MCPyV tLTAg, and p53 (Figure 1C), none of which were detectable at appreciable levels in control hair follicles (Supplemental Figure 3). While a reliable sTAg antibody is not available, the sTAg target REST corepressor 2 (RCOR2) (31) was also detected in the atypical cellular aggregates (Supplemental Figure 4A). Although microscopic cellular aggregates with KRT8^+^ cells could be detected at all time points examined between 2 weeks and 12 months after transgene induction, progression to gross tumors resembling MCCs was not detected in *SLA* mice (*n* = 15). These findings suggest that expression of MCPyV TAgs together with exogenous ATOH1 in epidermal cells located specifically near the hair follicle stem cell niche is sufficient to initiate tumorigenesis, but fails to drive progressive growth and formation of grossly evident MCCs.

**Figure 2:**
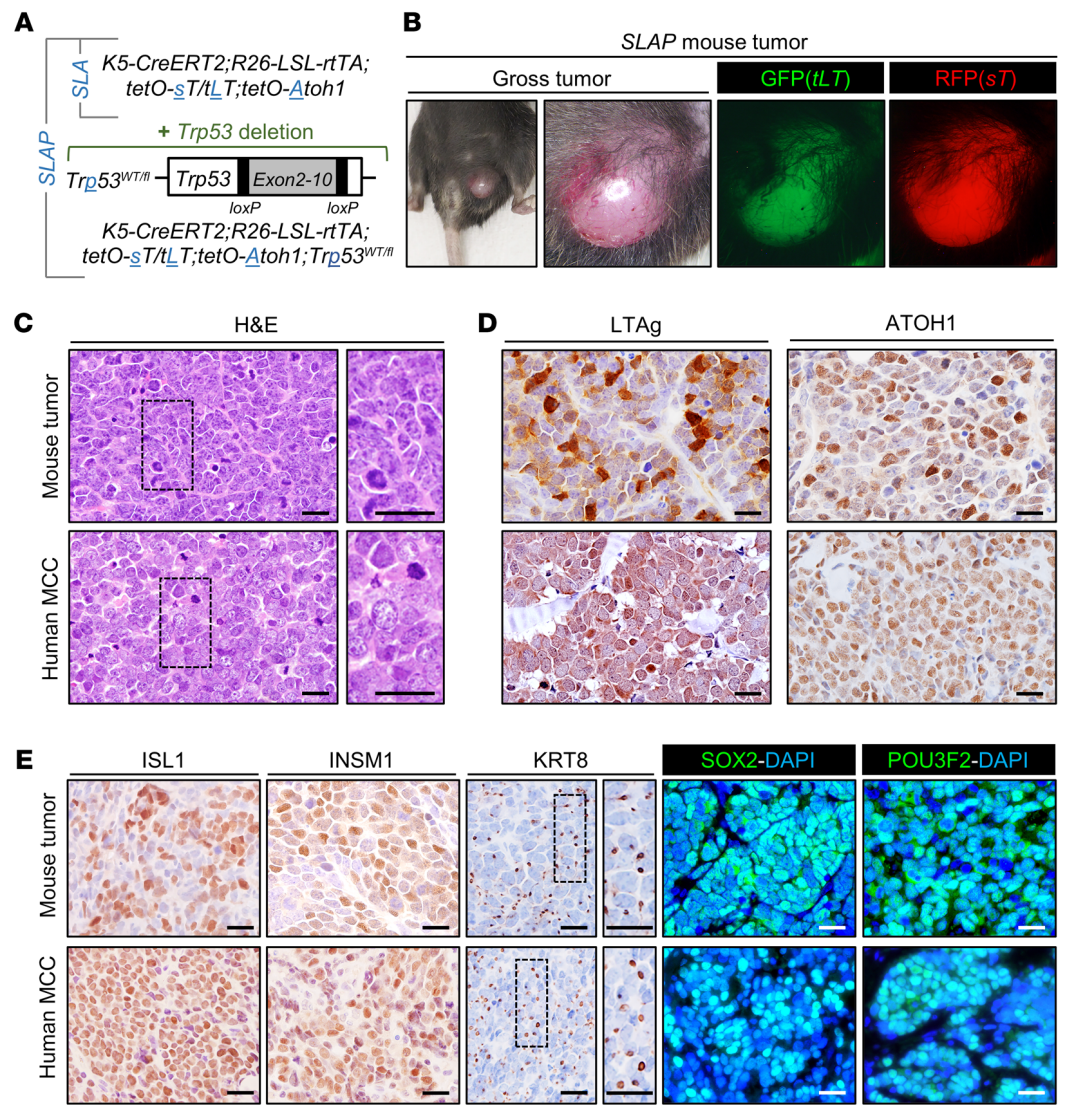
In vivo reprogramming using ATOH1 in p53-deficient cells enables development of full-blown murine MCC. (**A**) Addition of conditional *Trp53* allele to generate *SLAP* mice expressing MCPyV sTAg, tLTAg, and ATOH1, which are also deficient in p53, in *Krt5*-expressing cells and their progeny. (**B**) Gross tumor arising in *SLAP* mouse 4 months after transgene induction. (**C**) Similar histopathology of *SLAP* mouse tumor and human MCC. Immunostaining for (**D**) transgene expression and (**E**) MCC marker expression. Scale bars: 25 μm.

The accumulation of p53 in nascent MCCs (Figure 1C) was unexpected, since MCPyV sTAg functionally inactivates p53 in human cells by increasing the levels of both MDM2 and CK1α, which activates MDM4 (32). Given the presence of apoptotic cells in the microscopic tumor-like aggregates in *SLA* mice (Figure 1C), we considered that the failed progression to full-blown MCC might be due at least in part to p53-mediated cell death. To explore this possibility, we next generated mice designated *SLAP*, which also carried 1 floxed p53 allele (*Trp53^WT/fl^*) (ref. 33 and Figure 2A) yielding cells hemizygous for *Trp53* following recombination. Six out of fourteen *SLAP* mice were euthanized for humane reasons, reflecting unanticipated morbidity also reported in other mouse models expressing MCPyV TAgs and deficient in p53 (12, 13). Each of the remaining 8 mice developed 1 or more grossly visible tumors resembling human MCCs between 11 and 22 weeks after transgene induction (Figure 2, B and C). Notably, the WT *Trp53* allele was lost in all MCCs for which DNA was available for analysis (*n* = 5) (Supplemental Methods and Supplemental Figure 5, showing absence of sequence from amplicons covering Trp53 exons 2-10), pointing to a requirement for complete loss of p53 for tumor expansion.

Ten of the eleven skin tumors arising in *SLAP* mice exhibited histologic features highly characteristic of human MCCs, including a monomorphous small blue cell phenotype, finely stippled chromatin, prominent mitoses, and nuclear molding (Figure 2C) (see Methods). Variable numbers of tumor cells also expressed tLTAg and ATOH1 (Figure 2D) as well as multiple protein markers detected in human MCCs, including ISL1, INSM1, SOX2, POU3F2, and KRT8, the latter in a dot-like pattern highly characteristic of MCC (Figure 2E). In addition, multiple sTAg target proteins (31) were detected by immunoblotting or immunostaining (Supplemental Figure 4). Despite tumor initiation from hair follicle epithelium (Figure 1, B and C), the mouse MCCs, like the great majority of human MCCs, were largely localized within the dermal compartment of skin without obvious connections to either the epidermis or hair follicles.

**Figure 3:**
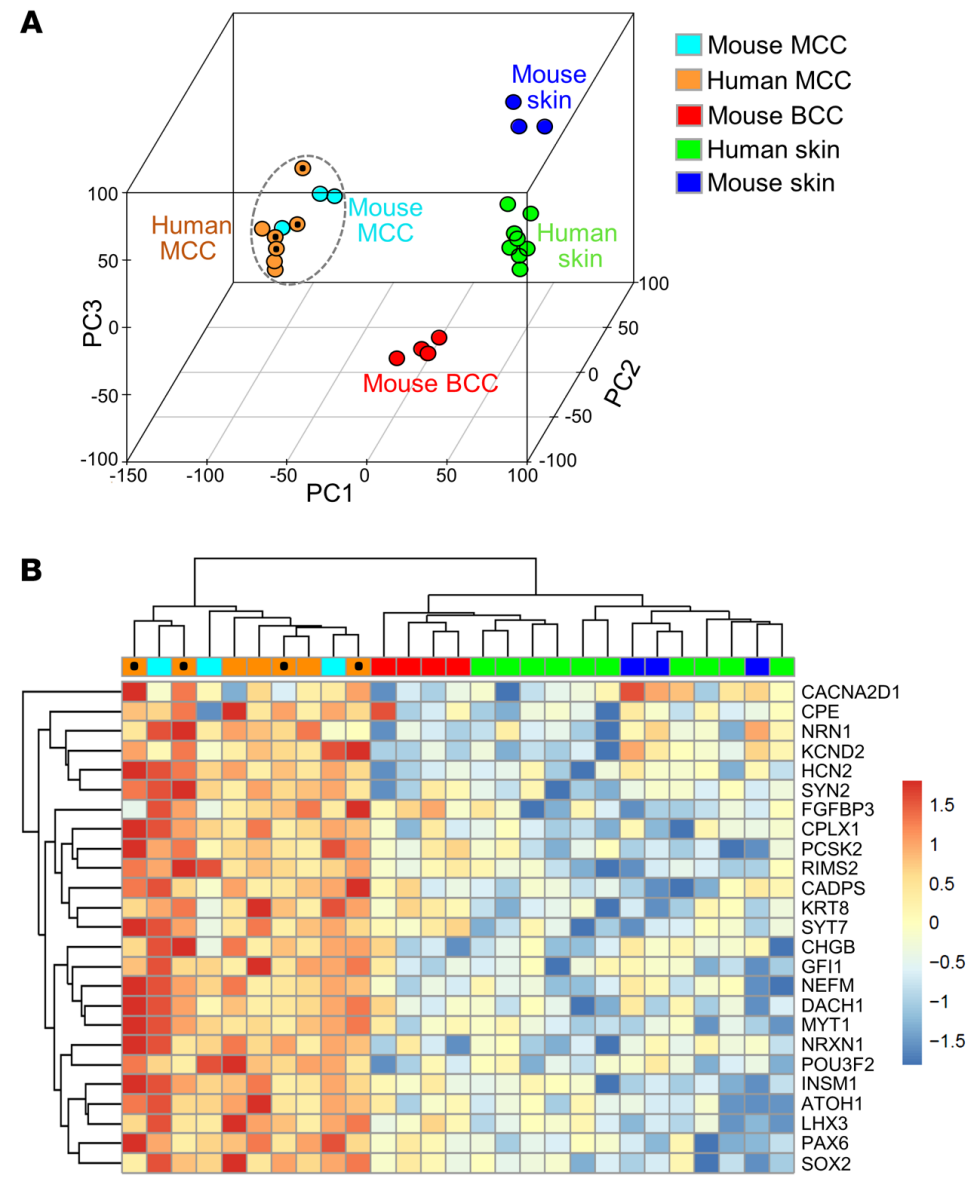
Cross-species transcriptome analysis of MCC. (**A**) Principal component analysis plot of global transcriptomes showing similarity of mouse (*n* = 3) and human (*n* = 7) MCCs, with a well-defined separation from normal mouse (*n* = 3) and human (*n* = 10) skin as well as mouse BCCs (*n* = 4). (**B**) Hierarchical clustering of transcripts enriched in normal mouse Merkel cells shows similar expression patterns in mouse and human MCCs. Data from MCPyV-positive human MCCs are marked with black circles.

To further investigate the similarity between human MCCs and MCC-like skin tumors arising in *SLAP* mice, we performed RNA-Seq on tumor specimens. We also compared the MCC tumor transcriptomes to those of normal mouse skin, normal human skin, and mouse basal cell carcinoma (BCC) (34), a common epithelial skin tumor. Principal component analysis revealed a high degree of similarity between mouse and human MCC tumor samples that clustered together; additionally, these samples clustered separately from normal mouse and human skin as well as mouse BCCs (Figure 3A). To better define the similarity among MCCs, we also generated a heatmap of pairwise Spearman’s correlations across tumor and skin transcriptomes (Supplemental Figure 6). Hierarchical clustering again grouped the mouse and human MCCs together, with high overall similarity among MCCs from both species based on an average Spearman’s correlation of 0.74. In addition, the scatter plot in Supplemental Figure 7 shows concordance of the most highly upregulated genes in MCCs of both species when compared with normal skin, and Supplemental Figure 8 shows pathway enrichment analysis comparing mouse MCCs to normal skin. Finally, examination of transcripts highly expressed in mouse Merkel cells (ref. 35 and Figure 3B) or neuroendocrine variants of lung, prostate, and bladder cancers (ref. 36 and Supplemental Figure 9) highlighted the molecular similarities of mouse and human MCCs to normal Merkel cells as well as neuroendocrine cancers arising in other organs, respectively.

Although multiple polyomaviruses infect humans (37), only MCPyV has been convincingly linked to a human cancer. Using direct in vivo cellular reprogramming with ATOH1, we have generated what we believe is the first adult murine model of MCC. Several of our findings are noteworthy. Despite the broad expression pattern of the *Krt5* promoter in skin epithelia, initiation of TAg-driven mouse MCCs in our model appears to occur in or near a restricted domain of the hair follicle that harbors several stem cell populations (30). This is of interest since MCPyV-positive human MCCs have a low burden of UV mutations, in keeping with a cell of origin that resides in deeper compartments of skin, including the hair follicle, rather than more superficial regions, such as the interfollicular epidermis (see Figure 1B). In addition, the hair follicle is a site of relative immune privilege (38), perhaps allowing for survival and expansion of viral antigen-expressing cells that may be eliminated if recognized as foreign in other regions of skin. Finally, stem or progenitor cells may have greater plasticity and thus be preferentially susceptible to ATOH1-mediated postnatal reprogramming into the Merkel cell lineage.

The requirement for *Trp53* deletion in our model is also of interest since *TP53* mutations are uncommon in MCPyV-positive human MCCs, perhaps because p53 is depleted due to sTAg-mediated upregulation of MDM2 and the MDM4 activator CK1α (32). However, efficient disruption of p53 function seems unlikely in our murine *SLA* model, given the accumulation of p53 in nascent tumors (Figure 1C). The differential requirement for loss of *Trp53* in viral TAg-driven mouse MCC, but not human MCC, may also be due to the striking divergence of p53-regulated target genes in mouse versus human cells (39). Our data argue that either functional inactivation of p53 in human MCCs or genetic deletion of *Trp53* in our mouse model is required for MCPyV-driven MCC tumorigenesis. Importantly, our immunostaining studies and transcriptomic data highlight the strong similarity between human and mouse MCCs despite the different mechanisms leading to inhibition of p53.

In summary, our findings underscore the utility of modulating cell fate to generate a neoplasm without a defined cell of origin; establish a pivotal role for MCPyV T antigens in the pathogenesis of virus-positive MCCs; demonstrate how tumors that appear to reside entirely within the dermis may originate from follicle epithelia; and set the stage for future studies centered on gaining deeper insight into MCC biology, mechanisms underlying viral TAg-driven tumorigenesis, and preclinical testing of novel therapeutics.

## Methods

### Mouse models

#### Transgenic mouse production.

Transgenic mice carrying doxycycline-inducible MCPyV T antigens and fluorescent reporters, designated *tetO-sT/tLT*, were generated by coinjection of *tetO-sTAg-IRES-RFP* (*tetO-sT*) and *tetO-tLTAg-IRES-GFP* (*tetO-tLT*) cassettes (Supplemental Figure 1A) into fertilized (C57BL/6 × SJL) F2 mouse oocytes by the University of Michigan Transgenic Animal Model Core. Details and characterization of *tetO-sT/tLT* transgenic mice are provided in Supplemental Methods and Supplemental Figure 1.

#### Generating mice with inducible transgene expression.

To generate a model that would allow tight control of transgene expression both spatially and temporally, we employed a triple-transgenic model that included (a) a hormone-inducible Cre allele, (b) a Cre-inducible rtTA allele, and (c) *tetO*-driven effector alleles. We employed the *K5-CreERT2* strain (26) to drive tamoxifen-inducible Cre activity in K5-expressing epidermal cells, including Merkel cell progenitors; B6.Cg-*Gt(ROSA)26Sor^tm1(rtTA,EGFP)Nagy^*/J mice (The Jackson Laboratory, stock no. 005670; ref. 27), designated *R26-LSL-rtTA*, to drive expression of rtTA in recombined cells and all of their progeny; and *tetO-sT/tLT* strains for expression of MCPyV sTAg and tLTAg in mice with a target genotype of *K5-CreERT2;R26-LSL-rtTA;tetO-sT/tLT* (*SL*). A *tetO-Atoh1* allele (28) was added to drive cells into the neuroendocrine lineage in *K5-CreERT2;R26- LSL-rtTA;tetO-sTAg/tetO-tLTAg;tetO-Atoh1* (*SLA*) mice. In mice treated with tamoxifen to activate Cre function, recombination at the ROSA locus leads to rtTA expression, and transgene expression is induced by doxycycline. To also alter *Trp53* gene dosage, mice were crossed with conditional B6.129P2-*Trp53^tm1Brn^*/J (*Trp53^fl/fl^)* mutant mice (The Jackson Laboratory, stock no. 008462; ref. 33) to generate *K5-CreERT2;R26-LSL-rtTA;tetO-sT/tLT;tetO-Atoh1;Trp53^fl/WT^* (*SLAP*) mice, enabling deletion of 1 copy of the *Trp53* gene after recombination. Additional details are provided in Supplemental Methods and Supplemental Table 1.

#### Transgene induction and tumor monitoring.

Transgene expression with or without *Trp53* deletion was induced in *SLA* (*n* = 15; 11F/4M) and *SLAP* (*n* = 14; 10F/4M) mice starting at P21–P24 by continuous administration of tamoxifen chow at 400 mg/kg in Teklad Global rodent diet (Envigo) and doxycycline (200μg/mL, Fisher Scientific) in drinking water containing 5% sucrose. Mice were monitored biweekly for skin phenotypes and tumor development. *SLA* mice were monitored for up to 12 months, with no apparent skin tumors developing during this time. *SLAP* mice were monitored until skin tumors developed or until euthanasia was required for humane reasons (*n* = 6/14), which in 2 mice included growth of grossly evident internal tumors that were not MCCs.

#### Tumor development.

Gross skin tumors (*n* = 11) arising between 11 and 22 weeks after transgene induction in *SLAP* mice (*n* = 8) were GFP^+^ and RFP^+^ and expressed ATOH1 and SOX2 as well as other markers, including ISL1, INSM1, POU3F2, and KRT8, in at least focal areas consistent with human MCC. All tumors except for one were scored as histologically consistent with human MCC by a board-certified dermatopathologist. The outlier arose on an ear at the site of an ear tag, was classified histologically as undifferentiated, and did not express appreciable levels of most MCC markers. Internal tumors with an undifferentiated phenotype were identified in 3 of 8 *SLAP* mice with cutaneous tumors. Basal cell carcinomas arising in *K5-Gli2* mice were harvested at 7 to 9 months of age.

Immunostaining, acquisition of human tissue, RNA isolation, and sequencing, and processing and analysis of human and mouse RNA-Seq data sets are described in Supplemental Methods.

### Study approval

#### Animal studies.

All mice were housed and maintained and procedures performed according to University of Michigan IACUC guidelines under animal protocol PRO00008710.

#### Human studies.

Human MCC tumor specimens were collected from patients according to protocol approved by the University of Michigan IRB (study IDs: HUM00050085 and HUM00046018). Normal skin punch biopsies (5 mm) from healthy volunteers were collected after informed, written consent under a protocol approved by the local ethics board at the University Hospital Schleswig-Holstein, Campus Kiel, Germany (reference: A100/12).

## Author contributions

AAD and MEV conceived, designed, and supervised the study. MEV, JJVG, JA, DW, HZ, MG, and TLS performed animal modeling experiments, including generation of transgenic mice. AAD, MEV, PWH, SYW, and IB analyzed the mouse data. KH, CJL, and AMU performed experiments for targeted DNA sequencing, including processing data and statistical analysis. PWH, MTP, SW, and LCT performed transcriptomic analysis of human and mouse data sets. MG, MCK, and PC contributed mice or mouse tumors. PWH, SW, JEG, AMU, AMC, and LCT contributed human MCC tumor or human skin data sets or analysis tools for cross-species transcriptome analysis or next-generation sequencing (NGS) analysis. AAD and MEV wrote the manuscript, and PWH, SW, LJS, JSR, SYW, IB, MC, AMU, and LCT provided edits or made significant conceptual contributions, with all authors reviewing and approving the final version.

## Supplementary Material

Supplemental data

## Version 1 02/10/2022

In-Press Preview

## Version 2 04/01/2022

Electronic publication

## References

[1] Harms KL (2016). Analysis of prognostic factors from 9387 merkel cell carcinoma cases forms the basis for the New 8th Edition AJCC staging system. Ann Surg Oncol.

[2] Colunga A (2018). Merkel cell carcinoma in the age of immunotherapy: facts and hopes. Clin Cancer Res.

[3] Feng H (2008). Clonal integration of a polyomavirus in human Merkel cell carcinoma. Science.

[4] Harms PW (2018). The biology and treatment of Merkel cell carcinoma: current understanding and research priorities. Nat Rev Clin Oncol.

[5] Becker JC (2017). Merkel cell carcinoma. Nat Rev Dis Primers.

[6] Starrett GJ (2017). Merkel cell polyomavirus exhibits dominant control of the tumor genome and transcriptome in virus-associated merkel cell carcinoma. mBio.

[7] Wong SQ (2015). UV-associated mutations underlie the etiology of MCV-negative merkel cell carcinomas. Cancer Res.

[8] Knepper TC (2019). The genomic landscape of merkel cell carcinoma and clinicogenomic biomarkers of response to immune checkpoint inhibitor therapy. Clin Cancer Res.

[9] Harms PW (2015). The distinctive mutational spectra of polyomavirus-negative merkel cell carcinoma. Cancer Res.

[10] Wendzicki JA (2015). Large T and small T antigens of Merkel cell polyomavirus. Curr Opin Virol.

[11] DeCaprio JA (2021). Molecular pathogenesis of merkel cell carcinoma. Annu Rev Pathol.

[12] Spurgeon ME (2015). Tumorigenic activity of merkel cell polyomavirus T antigens expressed in the stratified epithelium of mice. Cancer Res.

[13] Shuda M (2015). Merkel cell polyomavirus small T antigen induces cancer and embryonic merkel cell proliferation in a transgenic mouse model. PLoS One.

[14] Verhaegen ME (2015). Merkel cell polyomavirus small T antigen is oncogenic in transgenic mice. J Invest Dermatol.

[15] Verhaegen ME (2017). Merkel cell polyomavirus small T antigen initiates merkel cell carcinoma-like tumor development in mice. Cancer Res.

[16] Sunshine JC (2018). Are there multiple cells of origin of Merkel cell carcinoma?. Oncogene.

[17] Moll I (2005). Human Merkel cells—aspects of cell biology, distribution and functions. Eur J Cell Biol.

[18] Woo SH (2015). Merkel cells and neurons keep in touch. Trends Cell Biol.

[19] Morrison KM (2009). Mammalian Merkel cells are descended from the epidermal lineage. Dev Biol.

[20] Van Keymeulen A (2009). Epidermal progenitors give rise to Merkel cells during embryonic development and adult homeostasis. J Cell Biol.

[21] Park HC (2012). Merkel cell carcinoma concurrent with bowen’s disease. Ann Dermatol.

[22] Falto Aizpurua LA (2018). A case of combined Merkel cell carcinoma and squamous cell carcinoma: Molecular insights and diagnostic pitfalls. JAAD Case Rep.

[23] Sirikanjanapong S (2010). Intraepidermal and dermal Merkel cell carcinoma with squamous cell carcinoma in situ: a case report with review of literature. J Cutan Pathol.

[24] Kervarrec T (2020). Polyomavirus-positive merkel cell carcinoma derived from a trichoblastoma suggests an epithelial origin of this merkel cell carcinoma. J Invest Dermatol.

[25] Ostrowski SM (2015). Ectopic Atoh1 expression drives Merkel cell production in embryonic, postnatal and adult mouse epidermis. Development.

[26] Indra AK (1999). Temporally-controlled site-specific mutagenesis in the basal layer of the epidermis: comparison of the recombinase activity of the tamoxifen-inducible Cre-ER(T) and Cre-ER(T2) recombinases. Nucleic Acids Res.

[27] Belteki G (2005). Conditional and inducible transgene expression in mice through the combinatorial use of Cre-mediated recombination and tetracycline induction. Nucleic Acids Res.

[28] Kelly MC (2012). Atoh1 directs the formation of sensory mosaics and induces cell proliferation in the postnatal mammalian cochlea in vivo. J Neurosci.

[29] Cotsarelis G (2006). Epithelial stem cells: a folliculocentric view. J Invest Dermatol.

[30] Jaks V (2010). The hair follicle-a stem cell zoo. Exp Cell Res.

[31] Park DE (2020). Merkel cell polyomavirus activates LSD1-mediated blockade of non-canonical BAF to regulate transformation and tumorigenesis. Nat Cell Biol.

[32] Park DE (2019). Dual inhibition of MDM2 and MDM4 in virus-positive Merkel cell carcinoma enhances the p53 response. Proc Natl Acad Sci U S A.

[33] Marino S (2000). Induction of medulloblastomas in p53-null mutant mice by somatic inactivation of Rb in the external granular layer cells of the cerebellum. Genes Dev.

[34] Grachtchouk M (2000). Basal cell carcinomas in mice overexpressing Gli2 in skin. Nat Genet.

[35] Hoffman BU (2018). Merkel cells activate sensory neural pathways through adrenergic synapses. Neuron.

[36] Balanis NG (2019). Pan-cancer convergence to a small-cell neuroendocrine phenotype that shares susceptibilities with hematological malignancies. Cancer Cell.

[37] DeCaprio JA, Garcea RL (2013). A cornucopia of human polyomaviruses. Nat Rev Microbiol.

[38] Paus R (2005). A ‘hairy’ privilege. Trends Immunol.

[39] Fischer M (2021). Mice are not humans: the case of p53. Trends Cancer.

