# Supplementary material for: Direct cellular reprogramming enables development of viral T antigen–driven Merkel cell carcinoma in mice
Source: J Clin Invest. 2022 Apr 1;132(7):e152069. doi: 10.1172/JCI152069 (PMC8970662; doi:10.1172/JCI152069)
Supplement: Supplemental data [file jci-132-152069-s038.pdf]

SUPPLEMENTAL MATERIALS
Supplemental Figures 1-9
Supplemental Tables 1-2
Supplemental Methods

Direct cellular reprogramming enables development of viral T antigen-driven Merkel cell carcinoma in mice

Monique E. Verhaegen,[1]* Paul W. Harms,[1,2,3,4] Julia J. Van Goor,[1] Jacob Arche,[1] Matthew T. Patrick,[1] Dawn Wilbert,[1] Haley Zabawa,[1] Marina Grachtchouk,[1] Chia-Jen Liu,[2,3] Kevin Hu,[5] Michael C. Kelly,[6] Ping Chen,[6] Thomas L. Saunders,[4,7] Stephan Weidinger,[8] Li-Jyun Syu,[1] John S. Runge,[1] Johann E. Gudjonsson,[1,9] Sunny Y. Wong,[1,4,10] Isaac Brownell,[11] Marcin Cieslik,[2,4,5] Aaron M. Udager,[2,4] Arul M. Chinnaiyan,[2,3,4,12,13] Lam C. Tsoi,[1,14] and Andrzej A. Dlugosz[1,4,10]*

Affiliations:
[1]Department of Dermatology,
[2]Department of Pathology,
[3]Michigan Center for Translational Pathology,
[4]Rogel Cancer Center,
[5]Department of Computational Medicine & Bioinformatics, University of Michigan, Ann Arbor, MI, USA
[6]Department of Cell Biology, Emory University School of Medicine, Atlanta, GA, USA
[7]Department of Internal Medicine, University of Michigan, Ann Arbor, MI, USA
[8]Department of Dermatology and Allergy, University Medical Center Schleswig-Holstein, Kiel, Germany
[9]A. Alfred Taubman Medical Research Institute,
[10]Department of Cell & Developmental Biology, University of Michigan, Ann Arbor, MI, USA
[11]Dermatology Branch, National Cancer Institute, Bethesda, MD, USA
[12]Howard Hughes Medical Institute,
[13]Department of Urology, and
[14]Department of Biostatistics, Center for Statistical Genetics, University of Michigan, Ann Arbor, MI, USA

MCK is currently at National Cancer Institute, Bethesda, MD, USA
PC is currently at Otogenetics, Atlanta, GA, USA

*e-mail: moniquev@umich.edu; dlugosza@umich.edu

Conflict of Interest: The authors have declared no conflict of interest exists.

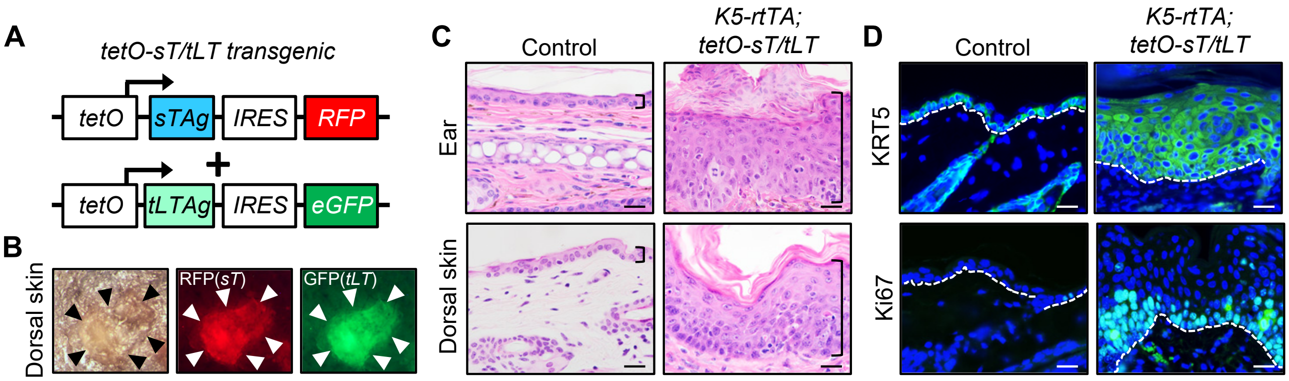

Supplemental Figure 1. Generation and validation of mice carrying *tetO*-regulated MCPyV sTAg and tLTAg. (A) Schematic of *tetO-sTAg-IRES-RFP* and *tetO-tLTAg-IRES-eGFP* cassettes used for co-injection to generate the *tetO-sT/tLT* transgenic mouse strains. **(B)** *K5-rtTA;tetO-sT/tLT* mice administered Doxycycline express MCPyV sT and tLT in *Krt5*-expressing epidermal cells. Brightfield and fluorescent images of dorsal skin 3 weeks after transgene induction. **(C)** Histology of ear and dorsal skin (2 weeks induction) shows a markedly hyperplastic epidermis (brackets) with dysplasia resembling squamous cell carcinoma *in situ*, as previously described in Cre-inducible MCPyV TAg models (Verhaegen ME *et al*., *J Invest Dermatol*. 2015. 135:1415-24; Spurgeon ME *et al*., *Cancer Res*. 2015. 75:1068-79). Scale bars: 25µm. **(D)** IF staining in dorsal skin shows expansion of basal layer (KRT5) and high proliferative index (Ki67) in epidermis of mice expressing MCPyV T antigens. Scale bars: 25µm.

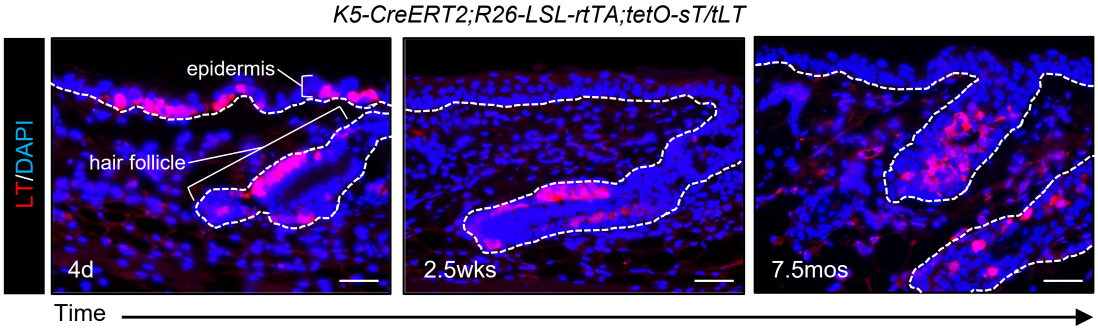

Supplemental Figure 2. Persistent expression of tLTAg in hair follicles of mice carrying *tetO*-regulated MCPyV sTAg and tLTAg. Fluorescent images of tLTAg (LT) staining in dorsal skin 4 days (4d), 2.5 weeks (wks) or 7.5 months (mos) after transgene induction in *K5-CreERT2;R26-LSL-rtTA;tetO-sT/tLT* mice. Over time, tLTAg expression is no longer detected in interfollicular epidermis but persists in hair follicles. Scale bars: 50μm.

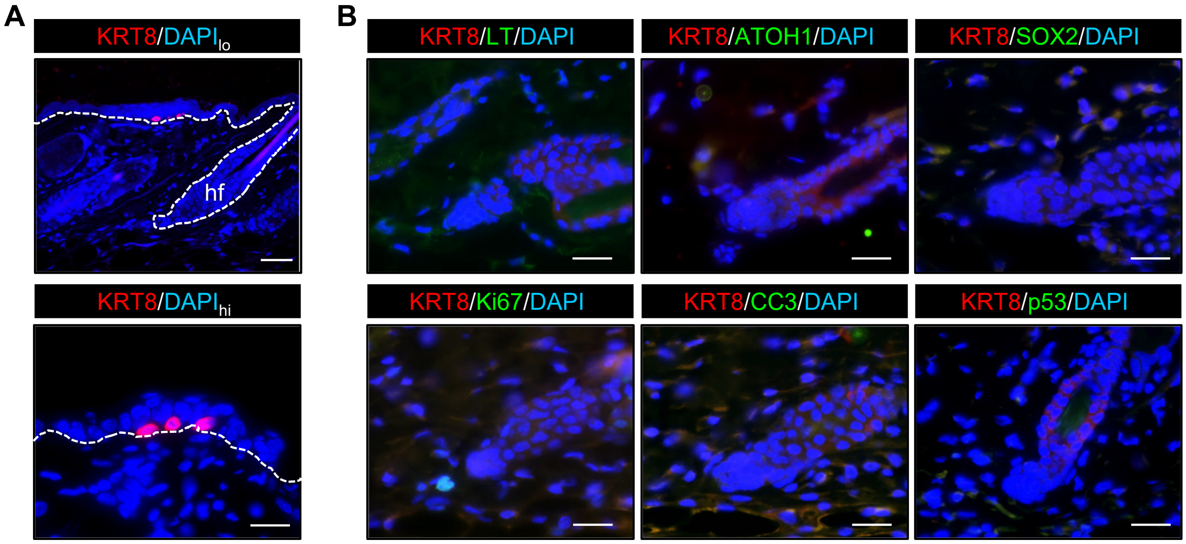

Supplemental Figure 3. Negligible expression of MCC markers, Ki67, CC3, and p53 in control skin. (A) Touch-dome associated Merkel cells in the interfollicular epidermis of control dorsal mouse skin are KRT8-positive (lo and hi magnification) (hf: hair follicle). Scale bars: upper panel 50µm, lower panel 25µm. **(B)** IF staining of control dorsal mouse skin for markers expressed in nascent follicle-associated tumors in *SLA* mice (see Fig. 1C). Scale bars: 25µm.

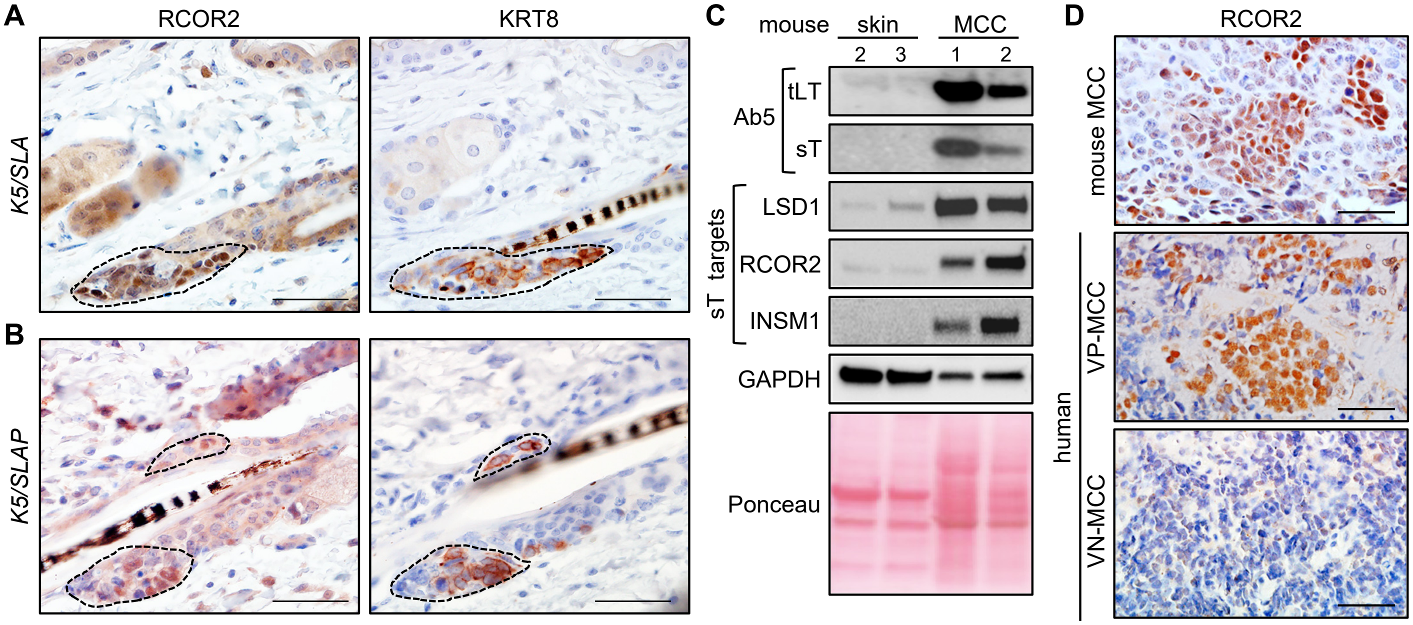

Supplemental Figure 4. Expression of MCPyV TAgs and sT targets in early nascent and full- blown mouse skin tumors. (A-B) Immunohistochemical staining for RCOR2 as a readout for sT expression in nascent follicle-associated tumors of dorsal skin in K5/*SLA* mice **(A)** or *K5/SLAP* mice **(B)** at 2 weeks post induction. KRT8 staining of nascent tumors is shown in adjacent tissue sections. **(C)** Immunoblotting of mouse dorsal skin or *K5/SLAP* mouse MCCs (matched to tissue used for RNA-seq studies) showing expression of MCPyV tLT and sT (Ab5), as well as downstream targets LSD1, RCOR2, and INSM1 activated by sT in complex with MYCL and EP400 (Park DE *et al., Nat Cell Biol.* 2020. 22:603-615). GAPDH and Ponceau stain are shown as protein loading controls. **(D)** IHC staining for sT target RCOR2 in a mouse SLAP tumor (mouse MCC) as well as MCPyV-positive (VP-MCC) and MCPyV-negative (VN-MCC) human MCCs. Scale bars: 50µm.

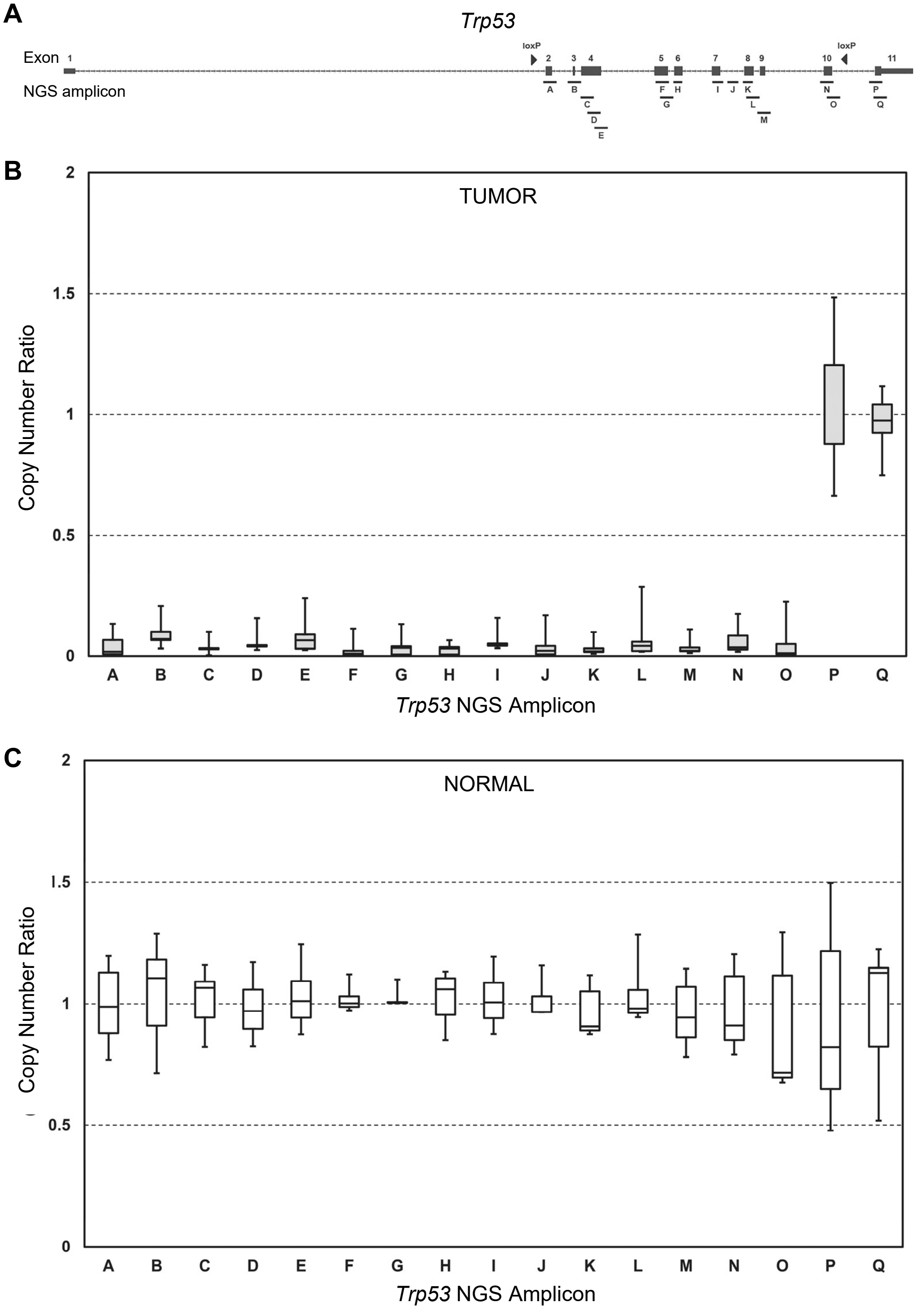

Supplemental Figure 5. Targeted next-generation sequencing (NGS) reveals two-copy *Trp53* loss in Merkel cell carcinoma (MCC) tumors from *SLAP* mice. (A) Scale diagram of *Trp53* locus with NGS amplicons (A-Q) targeting exons 2-11 and design of the conditional *Trp53* allele ($p53^{flox}$) with loxP sites flanking exons 2-10. **(B)** Box plots showing normalized copy number ratio (CNR) for each NGS amplicon (A-Q) across the *Trp53* locus for five MCC tumors from *SLAP* mice highlights two-copy loss of exons 2-10 (CNR < 0.25 for amplicons A-O) with two intact copies of exon 11 (CNR ~ 1 for amplicons P and Q), as expected based on the *Trp53* targeting design. **(C)** In contrast, box plots of amplicon-level normalized CNR for normal liver tissue (N=3) show two intact copies of all exons (CNR ~1 for amplicons A-Q).

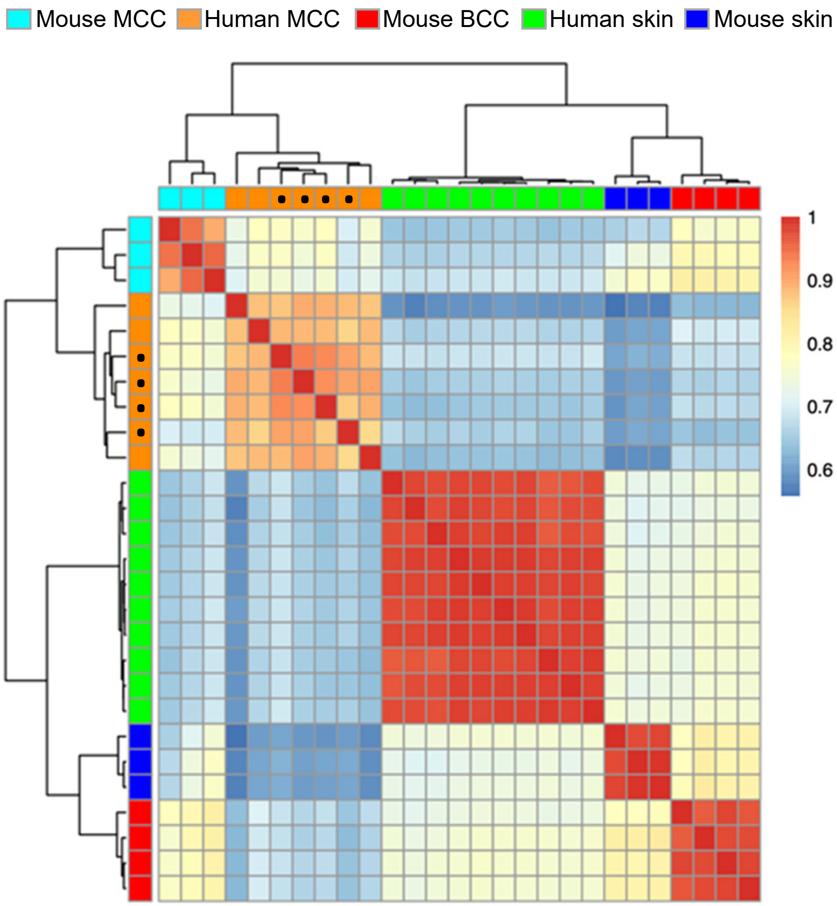

Supplemental Figure 6. Heatmap of pairwise spearman correlations across the indicated tumor and skin transcriptomes. Samples are ordered through unsupervised hierarchical clustering. Transcriptomes from human and mouse MCC tumors form a distinct branch (cluster). The similarity among MCCs from both species is high with an average Spearman correlation of 0.74, with the similarity among mouse MCCs higher than that among human MCCs (average Spearman correlations of 0.93 versus 0.89, respectively). Datasets from MCPyV+ human MCCs are marked (•).

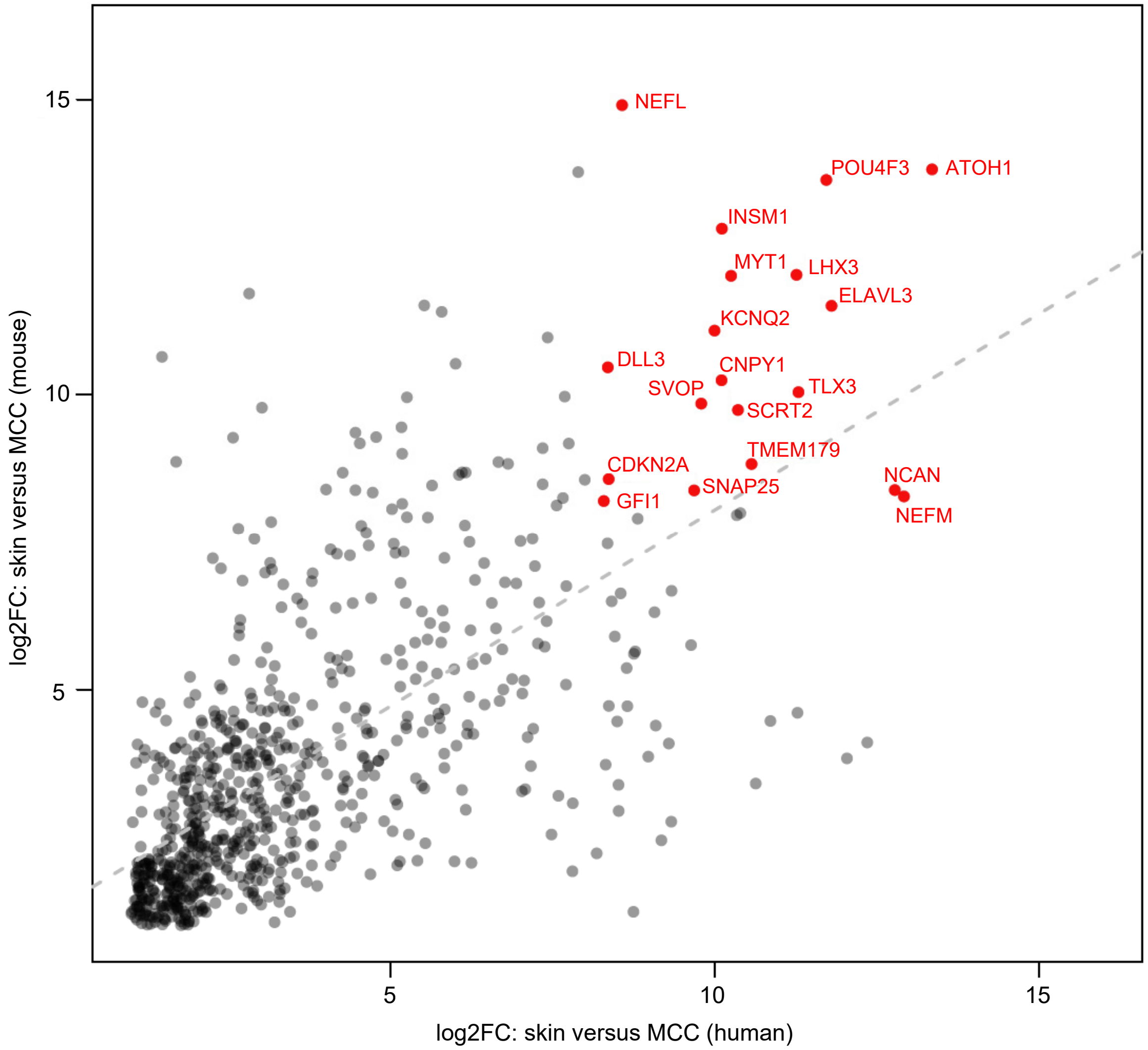

Supplemental Figure 7. Concordance of effect sizes (log2 fold-changes) of the skin vs MCC differential expression analyses in human (x-axis) and mouse (y-axis) for the common up-regulated genes. Genes with the largest fold changes in both organisms are shown in red.

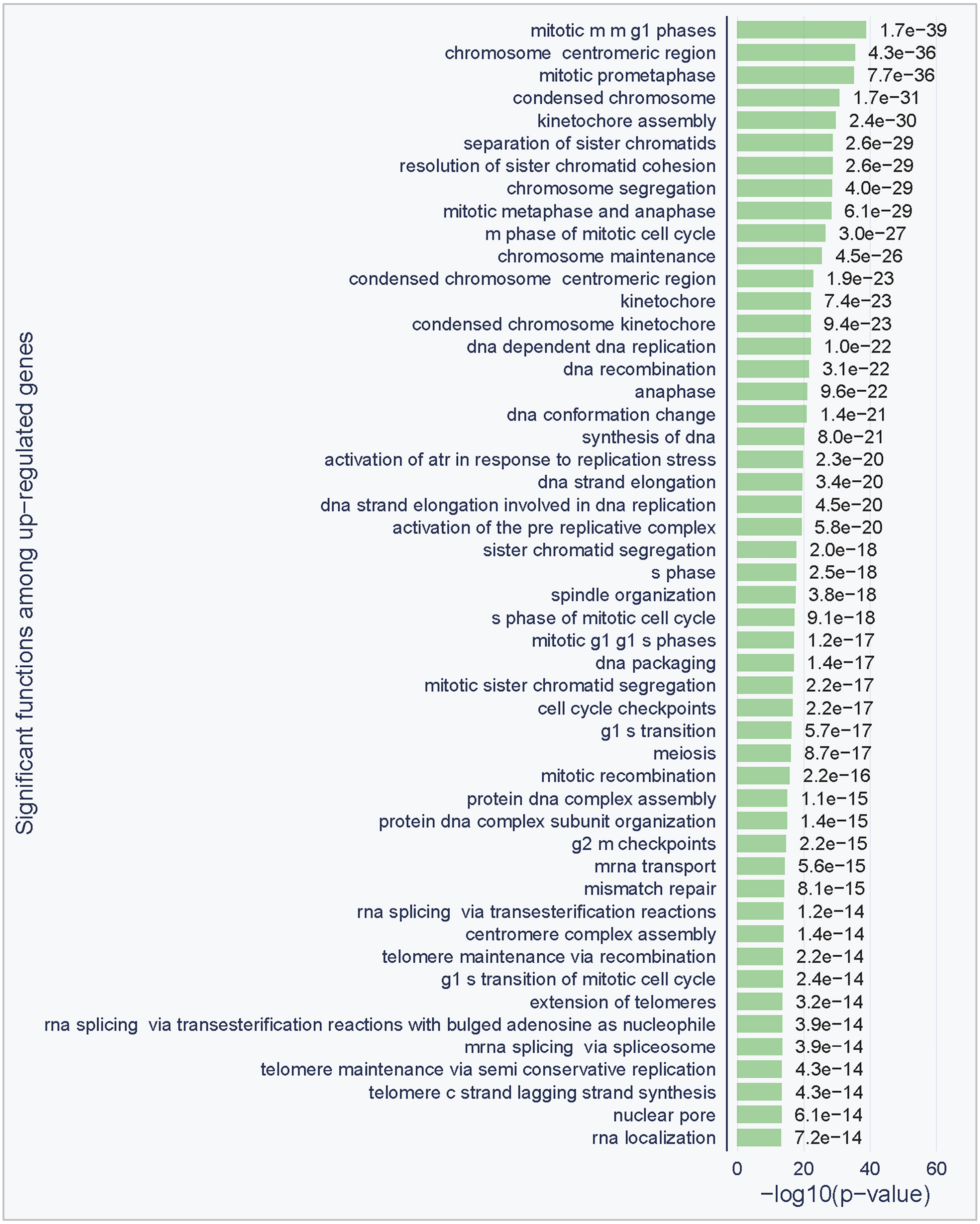

Supplemental Figure 8. Pathway enrichment analysis of genes significantly upregulated in mouse MCC tumors relative to normal mouse skin. The most significantly enriched pathways (false discovery rate ≤ 5%; observed-to-expected ratio ≥ 2) are shown, with P-values adjusted for multiple hypothesis testing.

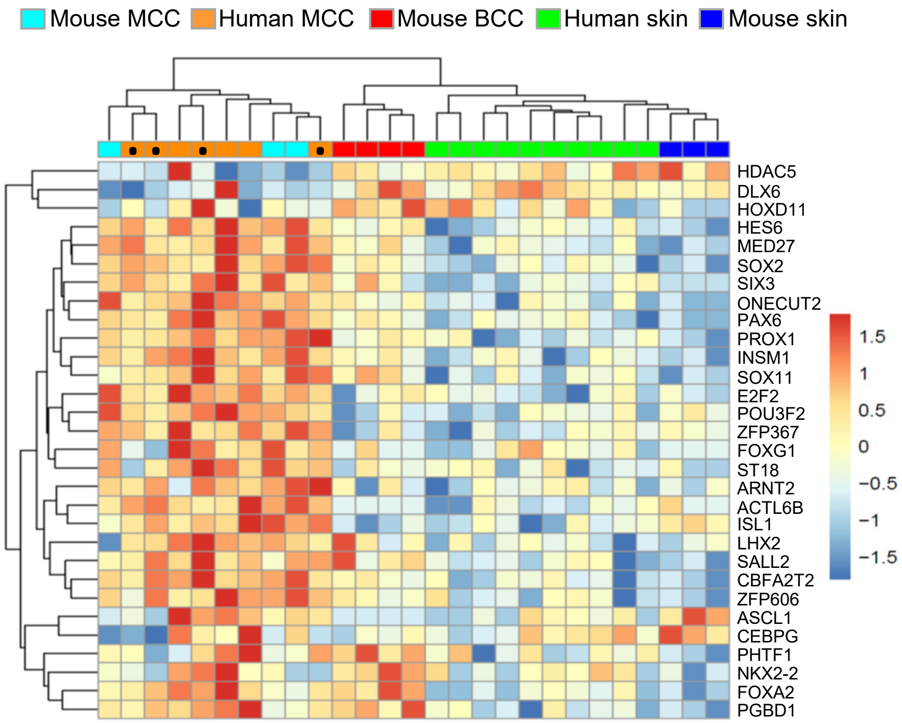

Supplemental Figure 9. Upregulation of signature neuroendocrine cancer transcription factors in human and mouse MCCs. Hierarchical clustering of transcripts encoding transcription factors enriched in small cell neuroendocrine cancers arising in lung, prostate, and bladder (Balanis NG *et al., Cancer Cell.* 2019. 36:17-34.e7), showing similarity of mouse (N=3) and human (N=7) MCCs, with a well-defined separation from normal mouse (N=3) and human (N=10) skin as well as mouse BCCs (N=4). Data from MCPyV+ human MCCs are marked (•).

Transgenic Line	Genotyping Primers	PCR product length
K5-CreERT2<br>*(K5-CreER)*	F:CATGCTTCATCGTCGGTCC<br>R:GATCATCAGCTACACCAGAG	412bp
B6.Cg-*Gt(ROSA)*<br>*26Sor*$^{tm1(rtTA,EGFP)Nagy}$/J<br>(*R26-LSL-rtTA)*<br>JAX Stock No. 005670	wt F:CGTGATCTGCAACTCCAGTC<br>wt R:GGAGCGGGAGAAATGGATATG<br>mut F:AAGTTCATCTGCACCACCG<br>mut R:TCCTTGAAGAAGATGGTGCG	wt=410bp<br>mut=173bp
tetO-sTAg-IRES-RFP +<br>*tetO-tLTAg-IRES-GFP*<br>*(tetO-sT/tLT)*	sT F:GGAATTGAACACCCTTTGGA<br>sT R:CTACAATGCTGGCGAGACAA<br>tLT F:CTGGGTATGGGTCCTTCTCA<br>tLT R:ATTGGGTGTGCTGGATTCTC	sT=237bp<br>tLT=155bp
tetO-Atoh1	F:CGCGCAATTAACCCTCACTA<br>R:CGGGAGAATGCAGCAGATAC	600-700bp
B6.129P2-*Trp53*^{tm1Brn}/J<br>(*p53*$^{fl/fl}$)<br>JAX Stock No. 008462	F:GGTTAAACCCAGCTTGACCA<br>R:GGAGGCAGAGACAGTTGGAG	wt=270bp<br>mut=390bp

Supplemental Table 1. Transgenic mouse lines. Mouse strains and genotyping primers used to generate *SLAP* mice. PCR for *Trp53* was carried out using Jackson Laboratory protocol 23419, Version 2.2. For all other reactions, the following PCR parameters were used: 95°C/5' denaturation; (95°C/30s-58°C/30s-72°C/30s) x35 cycles; 72°C/2' extension.

Antigen	Vendor; Cat No.	IHC/IF Dilution	IB Dilution
ATOH1	Proteintech; 21215	1:1000	
Cleaved Caspase 3 (CC3)	Cell Signaling Technology; 9661	1:300	
Cytokeratin 8 (KRT8)	Developmental Studies Hybridoma Bank; TROMA-I	1:500	
Cytokeratin 5 (KRT5)	Covance; PRB-160P	1:5000	
GAPDH (6C5)	Santa Cruz Biotechnology; 32233		1:1000
Ki67 (SP6)	Thermo Fisher Scientific; RM-9106-R7	1:300	
INSM1	Santa Cruz Biotechnology; 271408	1:200	1:300
ISL1	Developmental Studies Hybridoma Bank; clone 40.2D6	1:300	
LSD1	Cell Signaling Technology; 2139		1:1000
MCPyV LTAg (CM2B4)	Santa Cruz Biotechnology; 136172	1:500	
MCPyV sTAg and LTAg (Ab5)	Gift from James DeCaprio; Dana-Farber Cancer Institute		1:500
TP53 (CM5)	Novocastra Laboratories; NCL-p53-CM5	1:200	
POU3F2	Cell Signaling Technology; 12137	1:300	
RCOR2	Proteintech; 23696	1:200	1:500
SOX2	Neuromics; GT15098	1:2000	

Supplemental Table 2. Source of primary antibodies and dilutions used for immunohistochemical (IHC) and immunofluorescent (IF) tissue staining or immunoblotting (IB).

Supplemental Methods

Mouse Models

Production of transgenic mice. tetO-sT/tLT mice carrying the doxycycline-inducible MCPyV T antigens and fluorescent reporters were created following co-injection of *tetO-sTAg-IRES-RFP (tetO-sT)* and *tetO-tLTAg-IRES-GFP (tetO-tLT)* (Supplemental Fig. 1A). The *tetO-sT* cassette, driving expression of sT and RFP (mCherry), was generated following *de novo* synthesis of a *sTAg-IRES-mCherry* cassette by Genscript USA (Piscataway, NJ), and contained a sT cDNA sequence (nucleotides: 199-756; GenBank Accession No. EU375803), followed by an encephalomyocarditis virus internal ribosome entry site (IRES) sequence (nucleotides: 2842-3416; pLVX-IRES-tdTomato plasmid from Clontech No.631238) and mCherry cDNA (nucleotides: 1-708; GenBank Accession No. AY678264). The *tetO-tLT* cassette, driving expression of a tumor-specific truncated 275aa LT antigen (1), was generated following *de novo* synthesis of a fragment containing cDNA sequence from MCCw168 (nucleotides: 1-234; join 666-1259; GenBank KC426954), followed by the same IRES sequence as above and eGFP cDNA (nucleotides: 97-816; GenBank Accession No. U55761). The *sT, tLT* and *eGFP* cDNAs contained Kozak sequences. Both *sT*- and *tLT*-containing fragments were flanked by *Sal1* and *EcoRV* sites for cloning into the pTet-Splice vector (Invitrogen), which contains a CMV promoter, regulatory sequences from the tet operon (*tetO*), an SV40 intron and poly(A) signal. The pTet-splice plasmid was modified to contain an additional *NotI* site in the pre-existing *XhoI* site (2) to allow for NotI digestion of both *tetO-sT* and *tetO-tLT* cassettes for co-injection into fertilized (C57BL/6 x SJL) F2 mouse oocytes by the University of Michigan Transgenic Animal Model Core. Founders that consistently transmitted both transgenes to progeny, presumably due

to co-integration at the same genomic locus, were identified by genotyping with primer sets specific for *sT* and *tLT* (Supplemental Table 1).

C*haracterization of tetO-sT/tLT transgenic mice.* Five independent *tetO-sT/tLT* lines were established and crossed to *K5-rtTA* “Tet-On” mice (3) for transgene-induction studies and phenotype screening. *K5-rtTA;tetO-sT/tLT* mice were induced at P21 with doxycycline chow (1g/kg) from Bio-Serv (Frenchtown, NJ) to drive expression of sT, tLT and fluorescent reporters RFP and GFP, respectively (Supplemental Fig. 1B). Three independent lines (*tetO-sT/tLT*^{224}, *tetO-sT/tLT*^{227}, and *tetO-sT/tLT*^{277}), when also carrying the *K5-rtTA* transgene and induced with doxycycline, yielded a strikingly hyperplastic epidermis with squamous cell carcinoma in situ-like alterations (Supplemental Fig. 1C, D), as seen in previous mouse models expressing either MCPyV sT, or sT and tLT, in epidermis (4, 5). These three lines were crossed to C57BL/6J breeders (The Jackson Laboratory, Stock No. 000664) to generate maintenance colonies.

Generation of *K5-CreERT2;R26-LSL-rtTA;tetO-sT/tLT;tetO-Atoh1;Trp53*$^{fl/WT}$(*SLAP*) mice for tumor development studies employed both the *tetO-sT/tLT*^{224} strain, which produced the most dramatic skin phenotype in initial screening with *K5-rtTA* driver (see above), as well as the *tetO-sT/tLT*^{227} strain. Mouse details and genotyping information are provided in Supplemental Table 1.

Tissue harvesting and processing. Dorsal skin biopsies (1.0 x 1.5cm) from *SLA* or *SLAP* mice were taken at 14 days post tamoxifen/doxycycline induction for early analysis, or at 10 or 16 weeks for later time points, prior to terminal harvest. All skin tumors arising in *SLAP* mice were

harvested prior to maximum allowable size (1.8cm x 1.8cm) between 11 and 22 weeks after transgene induction. Matched control littermates were also harvested at similar times. For all procedures, mice were anesthetized with isoflurane gas or a mixture of ketamine-xylazine, and final euthanasia performed using carbon dioxide. Brightfield and fluorescence imaging was performed with a Leica MZFL II dissecting microscope with an Olympus DP71 digital camera and Olympus cellSens Standard imaging software. All harvested tissue was maintained in chilled Hank's Balanced Salt Solution during harvest, fixed in 10% neutral buffered formalin overnight, transferred to 70% ethanol and processed for paraffin embedding. Skin or tumor samples for RNA isolation were flash frozen in liquid nitrogen and stored for later use.

Acquisition of Human Tissue

Human MCC tumor specimens were collected from patients following written consent for use of surplus tissue according to a protocol approved by the University of Michigan Institutional Review Board (IRB Study ID: HUM00050085). Tissue was collected in chilled transport media (Dulbecco's Modified - Eagle's Medium containing 500U/500µg penicillin/streptomycin per mL media) and fixed overnight in 10% neutral buffered formalin, transferred to 70% ethanol and processed for paraffin embedding. The collection, processing, and sequencing of human MCC tumor specimens used for RNA-Seq has been previously described and includes samples from the Michigan Medicine Cutaneous Surgery and Oncology Program, and the MI-ONCOSEQ protocol for integrative tumor sequencing (IRB Study ID: HUM00046018) (9). Normal skin punch biopsies (5mm) from healthy volunteers were collected after informed written consent under a protocol approved by the local ethics board at the University Hospital Schleswig-

Holstein, Campus Kiel, Germany (Reference: A100/12) and preserved using PAXgene® tissue containers following the manufacturers specifications (9).

Tissue Immunostaining

Tissue in paraffin blocks was sectioned at 4-5µm, deparaffinized, and rehydrated for immunohistochemical (IHC) or immunofluorescent (IF) staining, prior to antigen retrieval in citrate-based buffer (0.01 mol/L citric acid, pH 6.8) for 15 min at 100° C. Quenching of endogenous peroxidases was performed with 3% H_2O_2 followed by blocking in 5% donkey serum for 1 hour. M.O.M. mouse Ig blocking reagent (Vector Laboratories, Burlingame, CA) was used for mouse primary antibodies on mouse tissue. All primary antibodies were incubated overnight in a humidified chamber at 4° C, with the exception of p53 co-staining which was carried out for 1hr at ambient room temperature. Details of primary antibodies including LTAg, ATOH1, cleaved caspase 3 (CC3), Keratin 8 (KRT8), Keratin 5 (KRT5), Ki67, ISL1, INSM1, SOX2, POU3F2, TP53, and RCOR2 are listed in Supplemental Table 2. Bound antibodies were detected with appropriate biotinylated or fluorophore-conjugated (AlexaFluor488, AlexaFlour594, or AlexaFluor555, from Jackson ImmunoResearch Laboratories or Invitrogen) secondary antibodies diluted at 1:300. IHC detection was carried out with the VectaStain ABC or M.O.M. Peroxidase Immunodetection Kits (Vector Laboratories) using SigmaFast diaminobenzidene as a substrate, followed by nuclear (DNA) counterstaining with hematoxylin and mounting with Permount (Thermo Fisher Scientific). IF-stained sections were mounted with ProLong Diamond or Gold Antifade with DAPI (Invitrogen) for visualization.

Tissue Immunoblotting

Total lysates from mouse dorsal skin (N=2) or *SLAP* tumors (N=2) was obtained by mechanical homogenization in RIPA buffer (Sigma, MO) supplemented with HALT phosphatase inhibitor cocktail (Thermo Scientific, IL) and Complete mini protease inhibitor cocktail (Roche, IN). Standard Bradford method using Bio-Rad Protein Assay dye reagent (Bio-Rad Laboratories, CA) was used for protein quantification and proteins were separated on 4-20% gradient SDS-polyacrylamide gels and transferred to Immobilon-P membranes (Millipore, MA). Membranes were stained with Ponceau for protein loading and transfer verification, followed by overnight incubation at 4ºC with primary antibodies including Ab5, GAPDH, LSD1, RCOR2, and INSM1. Antibody details are described in Supplemental Table 2. Detection was carried out with SuperSignal West Pico chemiluminescent substrate (Thermo Scientific, IL).

RNA Isolation and Sequencing

Mouse samples. Frozen tumor samples from a *SLAP* mouse (N=3 tumors) were disrupted and homogenized in QIAzol Lysis Reagent (Qiagen) with a 5-mm stainless steel bead in a TissueLyser II (Qiagen). RNA purification of tissue lysates was performed with the miRNAeasy Kit (Qiagen) with DNase I digestion as per manufacturer's instructions. RNA integrity was verified (RIN> 7) on an Agilent 2100 Bioanalyzer (Agilent Technologies, Santa Clara, CA) prior to RNA-seq library preparation from stranded mRNA following poly-A enrichment. The RNA-seq samples were sequenced on the NextSeq platform, producing paired-end reads (2x76bp) according to the manufacturer's protocol (Illumina, San Diego, CA).

Human samples. For human MCC tumors (N=7), previously reported transcriptome sequencing data were re-analyzed, with RNA extracted from flash-frozen human tumors as described (9). Four of seven human MCC tumors were MCPyV+ and wild-type for *TP53*. The three remaining MCCs were MCPyV-. Two of these tumors were sequenced and found to carry *TP53* mutations, whereas the *TP53* status of the remaining tumor is not known. RNA-seq libraries were then prepared from stranded mRNA following poly-A enrichment, and paired-end libraries were sequenced with the Illumina HiSeq 2000. Data for previously sequenced (MI-ONCOSEQ) human MCC tumors are deposited in dbGaP (accession: phs000673.v1.p1). For human control skin (N=10), previously published RNA-seq data (9) were re-analyzed (data can be accessed in GEO121212). RNA samples were prepared for sequencing using the Illumina Truseq® Stranded total RNA Protocol in combination with the RiboZero rRNA removal Kit and sequenced on the HiSeq2500.

Transcriptome analysis

RNA-seq reads were adaptor trimmed and mapped to the mouse reference genome (mm10) using STAR (6), and the number of uniquely mapped reads for each gene was counted using HTSeq (7) using GENCODE M18. Only genes with an average ≥1 read/sample were used in our subsequent analysis. All RNA-seq data generated in this study have been made available through the Gene Expression Omnibus (GEO) repository under the accession number GSE166751. The human RNA-seq data was processed analogously, using the GRCh37 as reference. DESeq2 (8) was used for sample normalization and modeling the read count. Pathway enrichment analysis was conducted using genes that are significantly differentially expressed (i.e., adjusted p-value ≤0.05; log2FC ≥ 1) in MCC when compared with normal skin. We used hypergeometric test to

evaluate the enrichment of differentially expressed genes among all gene ontology/KEGG pathways that are annotated with at least 20 and at most 200 genes.

Cross-species transcriptome analysis. R package biomaRt (10) was used to identify homologous genes between the 2 species, and only genes common to both species were used in the cross-species analysis. For cross-species comparison, quantile normalization was first applied to the transcriptomes across all samples, and inverse normalization was used to standardize gene expression values for result illustration in the heatmaps.

Targeted DNA sequencing

Approximately 2-3 mm^3 of frozen *SLAP* skin tumors (N=5) and normal liver from control mice (N=3) were disrupted manually with disposable pellet pestles using reagents and protocols supplied with the Qiagen DNeasy Blood and Tissue Kit for isolation of genomic DNA. Barcoded next-generation sequencing (NGS) libraries were generated from up to 20ng of DNA using the Ion AmpliSeq Library Kit 2.0 (Life Technologies, Carlsbad, CA) and a custom AmpliSeq DNA panel (Thermo Fisher Scientific) that targets the complete coding regions of 32 genes, including *Trp53* (11), prior to sequencing with the Ion Torrent NGS System. NGS reads were processed using the Ion Torrent Suite software and extensively validated in-house bioinformatics pipelines, and gene-level copy number ratios for Trp53 were generated, as described previously (11).

References

1. Shuda M, et al. T antigen mutations are a human tumor-specific signature for Merkel cell polyomavirus. *Proc Natl Acad Sci U S A.* 2008;105(42):16272-7.
2. Grachtchouk M, et al. Basal cell carcinomas in mice arise from hair follicle stem cells and multiple epithelial progenitor populations. *J Clin Invest.* 2011;121(5):1768-81.
3. Diamond I, et al. Conditional gene expression in the epidermis of transgenic mice using the tetracycline-regulated transactivators tTA and rTA linked to the keratin 5 promoter. *J Invest Dermatol.* 2000;115(5):788-94.
4. Verhaegen ME, et al. Merkel cell polyomavirus small T antigen is oncogenic in transgenic mice. *J Invest Dermatol.* 2015;135(5):1415-24.
5. Spurgeon ME, et al. Tumorigenic activity of merkel cell polyomavirus T antigens expressed in the stratified epithelium of mice. *Cancer Res.* 2015;75(6):1068-79.
6. Dobin A, et al. STAR: ultrafast universal RNA-seq aligner. *Bioinformatics.* 2013;29(1):15-21.
7. Anders S, et al. HTSeq--a Python framework to work with high-throughput sequencing data. *Bioinformatics.* 2015;31(2):166-9.
8. Love MI, et al. Moderated estimation of fold change and dispersion for RNA-seq data with DESeq2. *Genome Biol.* 2014;15(12):550.
9. Tsoi LC, et al. Atopic Dermatitis Is an IL-13-Dominant Disease with Greater Molecular Heterogeneity Compared to Psoriasis. *J Invest Dermatol.* 2019;139(7):1480-9.
10. Drost HG, and Paszkowski J. Biomartr: genomic data retrieval with R. *Bioinformatics.* 2017;33(8):1216-7.

11. McCool KW, et al. Murine Oviductal High-Grade Serous Carcinomas Mirror the Genomic Alterations, Gene Expression Profiles, and Immune Microenvironment of Their Human Counterparts. *Cancer Res.* 2020;80(4):877-89.
